# Discovery, Pathogenesis, and Complete Genome Characterization of *Lates calcarifer* Herpesvirus

**DOI:** 10.3390/genes15030264

**Published:** 2024-02-20

**Authors:** Bartjan Simmelink, Jordy P. M. Coolen, Wannes Vogels, Martin Deijs, Jessica L. M. van der Last-Kempkes, Kah Sing Ng, Siow Foong Chang, Koen Gevers, Liesbeth Harkema, Lia van der Hoek, Ad de Groof

**Affiliations:** 1 Department Discovery & Technology, MSD Animal Health, Wim de Körverstraat 35, P.O. Box 31, 5830 AA Boxmeer, The Netherlands; bartjan.simmelink@merck.com (B.S.); wannes.vogels1@merck.com (W.V.); jessica.van.der.last@merck.com (J.L.M.v.d.L.-K.); koen.gevers@merck.com (K.G.); 2 Department R&D-IT, MSD Animal Health, Wim de Körverstraat 35, P.O. Box 31, 5830 AA Boxmeer, The Netherlands; jordy.coolen@merck.com; 3 Laboratory of Experimental Virology, Department of Medical Microbiology and Infection Prevention, Amsterdam UMC, University of Amsterdam, Meibergdreef 9, 1105 AZ Amsterdam, The Netherlands; m.deijs@amsterdamumc.nl (M.D.); c.m.vanderhoek@amsterdamumc.nl (L.v.d.H.); 4 Amsterdam Institute for Infection and Immunity, Postbus 22660, 1100 DD Amsterdam, The Netherlands; 5 MSD Animal Health Innovation Pte Ltd., 1 Perahu Road, Singapore 718847, Singapore; kah.sing.ng@msd.com (K.S.N.); chang_siow_foong@nparks.gov.sg (S.F.C.); 6 Department Animal Research & Pathology, MSD Animal Health, Wim de Körverstraat 35, P.O. Box 31, 5830 AA Boxmeer, The Netherlands; l.harkema@gddiergezondheid.nl

**Keywords:** *Lates calcarifer*, Barramundi, *Alloherpesviridae*, *Ictalurivirus*, *Lates calcarifer* herpesvirus, phylogeny, VIDISCA-NGS, complete genome, virus culture, Koch’s postulates

## Abstract

In 2015 and 2016, two Barramundi (*Lates calcarifer*) farms in Singapore reported a disease outbreak characterized by lethargic behavior, pronounced inappetence, generalized skin lesions, erosions of the fins and tail, and ultimately high mortality in their fish. Next-generation sequencing and PCR confirmed presence of a novel virus belonging to the *Alloherpesviridae* family, *Lates calcarifer* herpesvirus (LCHV), which was subsequently isolated and cultured. We characterize, for the first time, the complete genome of two cultured LCHV isolates. The genome contains a long unique region of approximately 105,000 bp flanked by terminal repeats of approximately 24,800 bp, of which the first 8.2 kb do not show any similarity to described genomes in the *Alloherpesviridae* family. The two cultured isolates share 89% nucleotide identity, and their closest relatives are the viruses belonging to the genus *Ictalurivirus*. Experimental infections using one of the cultured LCHV isolates resulted in identical clinical signs as originally described in the index farm, both in intraperitoneal-injection infected fish and cohabitant fish, with mortality in both groups. Histopathological analysis showed pronounced abnormalities in the gills. Virus culture and PCR analysis confirmed the replication of LCHV in the infected fish, and thus Koch’s postulates were fulfilled.

## 1. Introduction

Barramundi/Asian sea bass/*Lates calcarifer* (hereafter called Barramundi) is a commercially important farmed catadromous fish in the Indo-West Pacific region that can be reared in freshwater and marine environments. Over the last decades, the fish has gained popularity in farming [1,2], but unfortunately the production of Barramundi is negatively affected by infectious diseases that cause great economic losses to farmers [3]. Fish die primarily in the grow-out phase from bacterial [3,4] or viral infections [5] or a combination of both [4,6]. Known viral pathogens are Red Seabream Iridovirus (RSIV), Infectious Spleen and Kidney Necrosis Virus (ISKNV), Scale Drop Disease Virus (SDDV), and *Lates calcarifer* Birnavirus (LCBV) [5,7,8]. In 2015, outbreaks with a distinct disease presentation were witnessed, which were suspectedly caused by a virus. Indeed, the presence of a novel virus belonging to the *Alloherpesviridae* family was described [9] and later also reported by other authors [10,11,12]. However, our understanding of the novel virus, *Lates calcarifer* herpesvirus (LCHV), is still incomplete. 

*Alloherpesviridae* belong to the order *Herpesvirales* that have fish and amphibians as their natural hosts [13,14]. The alloherpesviruses are mostly epitheliotropic [13]. Histological changes observed in diseased fish depend on the alloherpesvirus type and include epithelial hypertrophy, syncytia formation, epidermal cell hypertrophy, epidermal or branchial hyperplasia, hyperplasia and papillomas, or renal adenocarcinoma [13]. Long-term latency, characteristic for herpesviruses, has been observed in carp infected with CyHV-3 [15]. The alloherpesviruses are generally host specific [13], as replication of the virus in vitro can, for most of them, only be established in cell lines from the host itself. 

The *Alloherpesviridae* family comprises four genera, *Ictalurivirus*, *Salmonivirus*, *Batrachovirus,* and *Cyprinivirus* [14]. The full genome sequence has been determined and annotated for Ranid herpesvirus 1, 2 [16], and 3 [17] (RaHV-1, RaHV-2, RaHV-3), Bufonid herpesvirus 1 [18] (BfHV-1), Anguillid herpesvirus 1 [19] (AngHV-1), Cyprinid herpesvirus 1, 2, and 3 [20] (CyHV-1, CyHV-2, CyHV-3), Acipenserid herpesvirus 1 (also called white sturgeon herpesvirus by Waltzek et al., 2023 [21]—AciHV-1), Lake sturgeon herpesvirus 1 and 2 [22,23] (LSHV-1 and -2), Sterlet herpesvirus [24] (StHV), Ictalurid herpesvirus 1 [25] (IcHV-1) and -2 [26] (IcHV-2), and Silurid herpesvirus 1 [27] (SiHV-1). Among the partially sequenced alloherpesviruses, we find Acipenserid herpesvirus 2 [28] (AciHV-2), with predominantly the core genes sequenced. The best-studied viruses among the fish alloherpesviruses are Ictalurid herpesvirus 1 (also known as channel catfish virus) and Cyprinid herpesvirus 3 (also known as koi herpesvirus) [13]. Both viruses cause devastating diseases in aquaculture, with high mortality [29,30].

Here, we present the complete annotated viral genomes of two LCHV isolates from two geographically separated farms in Singapore. We also give a full overview of virus isolation, experimental infection, and initial histopathological analyses performed under experimental conditions for one of these two isolates. Importantly, a virus culture method was established to obtain pure stocks of the virus, and experimental infection with the isolated virus led to the reproduction of the disease signs and fulfillment of Koch’s postulates.

## 2. Materials and Methods

### 2.1. Barramundi Sample Collection and Preparation

#### 2.1.1. Field Sampling Sets

Diagnostic sampling sets were obtained from field farms for the purpose of health control or diagnostics. Sets were composed of variable (up to 12) pooled samples of serum and/or organs (kidney, spleen, liver) of Barramundi. Thirteen sampling sets were obtained from 6 farms in which Barramundi exhibited lethargy, inappetence, skin lesions, and discoloration (period: April 2015–September 2016). Five additional sampling sets of clinically healthy Barramundi that were submitted for a health check prior to the date of the first outbreak were tested as negative control samples (collected from 2 farms between October 2013 and January 2015). The total of 18 sampling sets originated from 8 farms in 5 countries (Indonesia, Saudi Arabia, Singapore, Sri Lanka, and Vietnam). 

Virus isolation and culture were performed on samples originating from two geographically separated farms in Singapore, and the isolates were designated V511 (July 2015) and V516 (January 2016).

#### 2.1.2. Field Sample Collection

Barramundi were anesthetized with AQUI-S^®^ (AQUI-S New Zealand Ltd., Lower Hutt, New Zealand) after a starvation time of 12–24 h. Barramundi were transferred to an anesthetic bath with 20 ppm AQUI-S^®^. If the fish were not in catatonic state within 2 min as checked with gentle pressure on the caudal fin, the AQUI-S^®^ concentration was increased with an additional 10–50 ppm. Fish were bled and blood was allowed to clot and transported refrigerated to the laboratory for processing the next day. Organs were removed, transported refrigerated to the laboratory, and stored upon arrival at −70 °C until further use.

#### 2.1.3. Field Sample Processing and Homogenization

Clotted blood samples were centrifuged at 13,500× *g* for 10 min at 4 °C. Serum was collected and stored at −70 °C until analysis. 

Organ samples were homogenized in a 1:9 ratio in Standard Vaccine Dilution Buffer (MSD Animal Health: composition Na_2_HPO_4_·2H_2_O 1.44 g/L; KH_2_PO_4_ 0.158 g/L; NaCl 15.0 g/L; KCl 0.20 g/L): 1 part of organ (weight) in 9 parts of buffer (volume). The homogenate was centrifuged 5500 rpm for 10 min at 4 °C. Supernatant was collected and stored at −70 °C until further use. 

### 2.2. Virus Culture on SBB Cells and Virus Titration

#### 2.2.1. Sea Bass Brain (SBB) Cell Culture

The Sea Bass Brain (SBB) cell line was derived from the brain of Barramundi, as described by Hasoon et al. [31] and Chi et al. [32] and was obtained from Dr. S. Koumans, MSD Animal Health, Department Bioprocess Technology and Support.

Cells were cultured in a CO_2_ incubator (Binder GmbH, Tuttlingen, Germany) at 28 °C and 5% CO_2_ in Eagle’s minimum essential medium (E-MEM) (Thermo Fisher Scientific, Bleiswijk, The Netherlands) containing 10% fetal calf serum (FCS (Moregate Biotech, Bulimba, Australia)). For sub-culturing, cells were washed with PBS (MSD Animal Health) and incubated 5 to 10 min in a 1% trypsin 0.02% Ethylene-diamine-tetra-acetic acid (EDTA) (Thermo Fisher Scientific, Bleiswijk, The Netherlands) solution at 28 °C for cell de-attachment. After re-suspending the cells in culture medium, cells were seeded at a density of 3.0 × 10^4^ cells/cm^2^. Cells were sub-cultured every 3 to 4 days.

#### 2.2.2. Virus Culture

Virus stocks were obtained upon passaging kidney homogenate of clinically affected fish on sea bass brain cells (SBB). The material was passaged 4 times and a tissue culture infective dose (^10^logTCID_50_) of 6.5/mL infectious dose was obtained for this Passage 4 batch. Aliquots of this stock were used as inoculum for virus growth experiments, as well as NGS-based virus discovery. Prior to inoculation, SBB cells were cultured overnight in a culturing flask at a density of 3.0 × 10^4^ cells/cm^2^. Culture harvest samples containing what appeared to be LCHV virus were diluted in culture medium to obtain an inoculum with a desired multiplicity of infection (MOI). The inoculum was added to the SBB cells after discarding the cell culture medium. After 60 min of incubation (28 °C, 5% CO_2_), the inoculum was discarded and replaced by fresh culture medium. Virus was harvested when a 100% cytopathic effect (CPE) was observed (2–6 days depending on the MOI, see formula below) by centrifuging all content for 5 min at 500× *g*. Supernatant was titrated immediately or stored at −70 °C until further use. All infection experiments and virus culture analyses were performed with virus stocks that were frozen and thawed once. We concluded from a duplicate experiment that freeze–thaw led to a 0.3 ^10^log TCID_50_ (factor of 2) reduction in viral titer compared to the freshly harvested virus sample.

MOI = (Amount of virus (mL) × ^10^log TCID_50_/mL)/(Total cells per flask)

#### 2.2.3. Endpoint Dilution/Titration Assays

Endpoint dilution assays were performed to determine the virus titer of samples by calculating the TCID_50_ according to the method of Reed and Muench [33]. SBB cells were cultured overnight in a 96-well plate with a density of 6.0 × 10^4^ cells per 100 µL per well to obtain a 60–75% confluent monolayer. Titration medium was prepared by a 1:1 dilution of SBB cell culture medium (Section 2.2.1) in EMEM medium. A ten-fold dilution series of harvested cultured virus was prepared in titration medium within a dilution range of 10^−2^ to 10^−7^. From each dilution, 10 wells per row of overnight cultured SBB cells in the 96-well plate were filled with 100 µL diluted virus sample and outer wells were filled with 100 µL titration medium as a negative control. The cells plus virus were incubated for a minimum of 6 days, after which the CPE was read and the TCID_50_ was calculated [33].

### 2.3. Library Preparation, NGS, and Phylogenetic Inference

#### 2.3.1. VIDISCA-NGS

Nucleic acids were isolated from pooled serum samples of sick fish of the index outbreak (July 2015), as well as CPE-causing cell culture supernatant from Passage 1 and 4 as described by Boom et al. [34], and VIDISCA-NGS (Virus discovery cDNA-AFLP using next-generation sequencing) was performed as described by de Vries et al. [35] with the following adaptations: Reverse transcription of RNA to cDNA was performed with Superscript II (200 U, Thermo Fisher Scientific, Bleiswijk, The Netherlands) and 2.5 µg non-ribosomal hexamers [36], in a mixture containing *E.coli* ligase (5 U, Invitrogen). In the subsequent digestion step, a single restriction enzyme was used (MseI, 10 U, New England Biolabs, Ipswich, MA, USA). AMPure XP beads were used to select for fragments of the proper size (between 100 and 400 bp). The DNA was quantified with the Qubit dsDNA HS Assay Kit (Thermo Fisher Scientific). A bioanalyzer (High Sensitivity DNA Analysis kit, Agilent Genomics, Santa Clara, CA, USA) was used to determine the average nucleotide length of the library. Following size determination and DNA copy calculations, 50 pM DNA were clonally amplified on beads using the Ion Chef System (Thermo Fisher Scientific). Sequencing was performed on an Ion Proton™ System (Thermo Fisher Scientific).

#### 2.3.2. Illumina Sequencing

Nucleic acids were isolated from 110 µL culture harvest of isolates V511 and V516 (Passage 4, Section 2.2.2) as described by Boom et al. [34]. Libraries for Illumina sequencing were prepared as described [37]. In short, dsDNA was randomly sheared using NEBNext dsDNA Fragmentase (New England Biolabs), followed by end repair with DNA Polymerase I, Large (Klenow) Fragment (New England Biolabs). An overhang was created with Klenow Fragment (3′-5′ exo-) (New England Biolabs) to which NEBNext Multiplex Oligos for Illumina adapters (diluted 1:1000) (New England Biolabs) were ligated with T4 DNA Ligase (5 U/µL) (Invitrogen). Short dsDNA fragments (<150 bp) and unbound adaptors were removed using AMPure XP beads. Ligated adaptors were treated with the USER enzyme (New England Biolabs) followed by a polymerase chain reaction (PCR) using Q5 High-Fidelity DNA Polymerase (NEB) and primers included in the Illumina adapter kit. The removal of post-PCR small fragments was performed with AMPure XP beads. The purified DNA was quantified with a Qubit dsDNA HS Assay Kit (Thermo Fisher Scientific). A bioanalyzer (High Sensitivity DNA Analysis kit, Agilent Genomics) was used to determine the average nucleotide length of the library. Paired-end sequencing, 2 × 150 bp, was performed on the Illumina MiSeq platform.

#### 2.3.3. Oxford Nanopore Sequencing

After thawing 1.0 mL of culture harvest of the LCHV isolates V511 and V516 (Passage 4, Section 2.2.2) showing 100% CPE, 50 µL 1M TRIS-HCl pH8.0, 2 µL 1M MgCl_2_, and 0.5 µL Benzonase (Sigma Aldrich, Darmstadt, Germany) were added and incubated for 4 h at 37 °C to reduce host cell DNA. Viral nucleic acid was extracted using the PureLink™ Viral RNA/DNA Mini Kit (Invitrogen cat. No. 12280050), according to the instructions of the manufacturer. Because of a low yield of extracted viral dsDNA, Lambda dsDNA (positive control in kit) was used to spike the LCHV dsDNA to reach the minimum required amount of nucleic acid for library preparation using the Rapid Sequencing Kit V14 (SQK-RAD114). Oxford Nanopore Technology (ONT) sequencing was performed on a MinION Mk1B using the Rapid Sequencing kit V14 on R10.4.1 flow cells using the default instructions of the manufacturer. Basecalling was performed with Guppy 6.4.6 using the super accurate model DNA_r10.4.1_e8.2_400bps_sup.cfg. Read statistics have been calculated using NanoComp 1.23.1 [38].

#### 2.3.4. Genome Assembly and Comparison

For V516, ONT reads were first aligned to the Escherichia phage Lambda (Accession Number J02459.1) using minimap 2.26-r1175 [39], and unmapped reads were subtracted using samtools prior to de novo assembly. For both LCHV virus isolates, de novo assembly was performed using Flye 2.9.2-b1786 [40] parameters (-g 160k -i 2 -m 1000 --meta). Additionally, for V511 and V516, the parameters –nano-hq and –nano-raw were added, respectively. The Illumina reads were aligned to the contig representing LCHV to detect genome termini by visual inspection and read depth distribution [41]. Homopolymer regions in the genomes were inspected, and based on Illumina reads manually corrected, using Geneious Prime 2022.1.1 (Geneious version 2022.1 created by Biomatters, available from https://www.geneious.com; accessed on15 February 2024). The two LCHV genomes of the isolates V511 and V516 were aligned in Geneious Prime 2022.1.1 using a global alignment according to the Needleman–Wunsch [42] cost matrix 93% identity using Geneious Prime 2022.1.1.

#### 2.3.5. Open Reading Frames Prediction and Annotation

The open reading frames (ORFs) in the final genomes were predicted and annotated using a custom process. In short, prokka 1.14.6 [43], eggnogmapper 2.1.12 [44], and orthofinder 2.5.5 [45] in which a set of 20 related alloherpesvirus genomes (see Supplementary Table S1) were used as a reference panel to predict and annotate ORFs. Eggnogmapper and orthofinder used diamond [46] with ultra-sensitive settings. The genome map was created using Geneious Prime 2022.1.1.

#### 2.3.6. Phylogenetic Inference

Twelve core genes were identified in V511 and V516 according to Walker et al. [22]. The amino acid sequences of the 12 core genes were concatenated and aligned using mafft v7.520 [47]. The phylogenetic tree was created using iqtree 2.2.2.7 [48] by first performing a best model fit followed by final tree creation using 1000-times ultrafast bootstrapping. The phylogeny based on the partial core genes was calculated identically. To visualize the trees, Figtree 1.4.4 (http://tree.bio.ed.ac.uk/software/Figtree/; accessed on 15 February 2024) was used.

### 2.4. Quantitative Polymerase Chain Reaction (qPCR)

#### 2.4.1. DNA Extraction

DNA extraction was performed using a MagNA Pure 96 System (Roche Diagnostics, Almere, The Netherlands) and a MagNA Pure 96 DNA and Viral NA Kit. Prior to extraction, 250 µL MagNA Pure 96 External Lysis Buffer was added to each 200 µL sample. DNA was isolated with a pre-installed external lysis protocol and eluted in 50 µL Milli-Q water. DNA was stored at −20 °C until further use.

#### 2.4.2. Primer and Probe Design

A screening method based on PCR (initially a nested PCR, later a qPCR) was designed on a part of LCHV ORF66 (DNA packaging terminase subunit 1, which is relatively conserved at the nucleotide level). These diagnostic PCRs were set up to enable the screening of sick fish and healthy controls and thus study the association between the presence of the virus and disease. The oligonucleotides (5′-3′) used to perform nested PCR diagnostics were LCHV-TER-FW: AGGGGCGGGATGAGATTAGA, LCHV-TER2-REV: AGGCTACAATTTCCGGGGTG, LCHV-TER3-FW: ATGCGAGGCTTTTTCATCGG, and LCHV-TER3-REV: TGATGGTCGTCTTACCGCAC. In a later phase, a quantitative PCR using matching oligonucleotide primers and a detection probe based on the C-terminal part of LCHV ORF66 (DNA packaging terminase subunit 1) were designed using Clone Manager 9. The following oligonucleotides and the probe were synthesized by Biolegio (Nijmegen, The Netherlands): LCHV-ORF66-FW: GGACCAATGTCAAAGTCGTG, LCHV-ORF66-REV: GTCGATCCTAGCAGGAAAGC, LCHV-ORF66-probe: 6FAM™-CGCGGGATGACCTCTTCTCG-TAMRA™ (see Supplementary Figure S1). The annealing of the primers and probe used for screening was verified on both V511 and V516 DNA.

#### 2.4.3. qPCR Master Mix and Program

Quantitative polymerase chain reactions (qPCRs) were performed using a CFX96 system (BioRad Laboratories, Lunteren, The Netherlands). Each reaction existed of 20 µL master mix containing 2 µL DNA template, 1× Probe Fast q-PCR Master Mix (KAPA), 200 nM forward primer, 200 nM reversed primer and 200 nM probe. Amplification occurred using a program starting at 95.0 °C for 3 min, followed by 40 repeats at 95.0 °C for 3 s and 60.7 °C for 30 s. A plate read was inserted at the end of each cycle. Each reaction was performed in duplicate, and all plates were spun 4 min at 2200× *g* before insertion in the CFX system.

#### 2.4.4. Conventional PCR and Gel Electrophoresis

Conventional PCRs were performed using a Veriti 96-well thermal cycler (Applied Biosystems, Bleiswijk, The Netherlands). A master mix was made containing 1× Supertaq buffer (Sphaero-Q), 0.02 U/µL Supertaq enzyme (Sphaero-Q), 0.2 mM deoxyribose nucleoside triphosphates (dNTPs–Sphaero-Q), and 1 µM forward and 1 µM reverse primer. For each sample, 2.0 µL DNA template was added in 48 µL PCR mix, and 2 µL sterile water was used as negative control. A PCR program was designed starting with 60 s initialization at 95 °C, followed by 40 repeats of denaturation, annealing and elongation for 30 s each at, respectively, 95 °C, 54 °C, and 72 °C. The program ended with a final elongation at 72 °C for 10 min. Samples were loaded with 1× ethidium bromide (ThermoFisher Scientific Bleiswijk, The Netherlands) in a 1.5% agarose gel (ThermoFisher Scientific Bleiswijk, The Netherlands) and 1× TAE buffer (ThermoFisher Scientific Bleiswijk, The Netherlands) and run at 115 volts for 60 min.

#### 2.4.5. Standards for Quantification

A dilution series containing a pUC57 vector with an LCHV-identity-sequence construct (synthesized by GenScript Biotech, Rijswijk, The Netherlands) was used as a positive control, indicator for efficiency and accuracy, and for sample quantification. The vector was dissolved and diluted in water in a range of 1.0 × 10^1^ copies/2 µL to 1.0 × 10^9^ copies/2 µL per qPCR reaction. The reference samples were included in all qPCR experiments and stored at −20 °C. Quantification of LHCV DNA in samples was performed by the supporting software (CFX-Manager version 3.1), which used the obtained data from the dilution samples to create a standard line.

### 2.5. Electron Microscopy

Copper grids of 400 mesh with a pure carbon film were exposed for 20 s to a glow discharge in air to make the film surface hydrophilic. A virus harvest (Passage 5) obtained 3 days after inoculation (12 mL) was 60 times concentrated using a Beckmann-Coulter ultracentrifuge at 30,000× *g* for 16 h at 4 °C. The virus was resuspended in 200 μL culture medium. Virus samples were placed on the carbon-coated grid with a volume of 10 µL and left to incubate for approximately 2 min. There was no pre-fixation applied, but uranyl acetate was added directly to the virus bound to the copper grid, after the removal of excess fluid containing concentrated virus. Excess sample was blotted with filter paper and 10 µL of water was placed on the grid and immediately removed again by blotting. Then, 10 µL of 1% uranyl acetate was placed on the grid for staining. After 30 s, excess stain was removed by blotting and the specimen was left to dry for a few minutes before viewing in the electron microscope. Specimen were observed in a JEOL 1011 transmission electron microscope operating at 80 kV. Images were recorded using a SIS Veleta 2k × 2k camera.

### 2.6. Experimental LCHV Infection in Barramundi

#### 2.6.1. Experimental Infection Sample Collection

The infectivity of the LCHV isolate V511 was experimentally tested in fish infection experiments at MSD Animal Health Innovations Pte Ltd. The governmental and institutional permissions to conduct such study are referenced in the ethics statement. Fish originating from a Singapore farm were screened for SDDV, LCHV, RSIV, *Tenacibaculum maritimum,* and *Streptococcus iniae* prior to commencement of the experiment. The aquarium system comprised a combination of both filtering and recirculation with the following parameters: salinity: 28 to 30 ppt; temperature: 30 ± 2 °C; ammonia: 0 ppm; nitrate: <150 ppm; nitrite: 0 to 0.05 ppm; pH: 7.0 to 8.0. Fish were fed EP2 pellets from Othohime (Japan).

For experimental infections, cell-free supernatant was prepared by centrifugation of the Passage 4 culture harvest of the LCHV isolate V511-infected SBB cells at 5500 rpm for 10 min at 4 °C following the development of complete CPE. Multiple aliquots of the harvest were stored at −70 °C for use in experimental infection experiments. The virus titer of the inoculum, which was back-titrated at each experiment, averaged 3.15 × 10^6^ TCID_50_/mL.

In a pilot experiment, a dose finding experiment was conducted. Two groups of five Barramundi were cohabitated in Tank 1, separated by netting. Tank 2 had a similar setup. Ten fish in the third tank served as a negative control group. The average weight of the fish was 15 grams (g) at the start of the experiment. Fish were starved for about 24 h prior to the infection to ensure the emptying of the gastro-intestinal tract to reduce the risk of injury and anesthetized (Section 2.1.2) before intra-peritoneal (IP, an iatrogenic route; midline between the base and tip of the pelvic fin) injection. Five fish in one partition of Tank 1 were IP infected with 0.1 mL of the LCHV isolate V511 virus harvested from tissue culture (Section 2.2.2). The cohabitant group in Tank 1 did not undergo experimental procedures and was supposed to undergo a natural infection in the tank. In Tank 2, five fish in one partition received a 10-times-diluted virus inoculum in vaccine dilution buffer (composition in Section 2.1.3) (IP, 0.1 mL). After a maximum holding time of 10 min, the fish were placed back in the assigned tank for recovery and observed for 21 days. Pooled serum was collected from cohabitant fish at the end of the experiment. 

After dose finding, the first main infection experiment was set up with two groups of 25 Barramundi that were cohabitated in the same tank, separated by netting. The average weight of the fish was 18 g at the start of the experiment. One group of fish was IP infected with the LCHV isolate V511 virus harvested from tissue culture (Section 2.2.2) but now IP injected with 0.3 mL per fish. During this experiment, samples were collected from the heart, spleen, kidney, skin, gills, brain, intestine, liver, and blood (serum). Samples from cohabitant fish were taken at 4, 7, 11, 14, and 18 days after the IP-infected group of fish was injected with the LCHV isolate V511. Organs and serum from IP-infected fish were collected at 17 days post-infection; only kidney samples were taken at 4, 7, 11, 14, and 18 days post-infection. Each sample consisted of the pooled organs of three randomly picked fish. Samples were stored at −70 °C. This experiment was terminated at Day 17 (IP group)–Day 18 (cohabitant group) upon a sharp increase in mortality which killed most of the fish or caused clinical signs.

A second main infection experiment was performed in 44 g fish, with a similar setup. One group of 20 fish was IP injected with 0.3 mL LCHV V511 per fish as described for the first main infection experiment, another uninfected cohabitant group of 30 fish were introduced into the adjacent tank partition separated by netting. At 7, 14, and 17 days post-infection, three fish were sampled for histopathology from the IP-infected group and at Day 7, 14, 17, and 21, five fish from the cohabitant group were sampled for virus isolation, assessment of serum and organs for virus culture on SBB cells, plus confirmatory PCR analysis. 

#### 2.6.2. Experimental Infection Sample Processing and Homogenization

Serum was obtained by allowing collected blood to clot and subsequently centrifugated at 13,500× *g* for 10 min at 4 °C and stored at −70 °C until DNA extraction. Organ samples were stored at −70 °C, thawed, and homogenized using a Precellys 24 Homogenizer instrument (Bertin Technologies, Montigny-le-Bretonneux, France). A 10% organ homogenate was prepared in phosphate-buffered saline (PBS) via one or two homogenization cycles of 20 s at 6500 rpm with a 10 s interval. Homogenization of the heart, spleen, kidney, brain, intestine, and liver samples was performed in one cycle, while skin and gill samples were homogenized twice. All samples were kept on ice during homogenization and were subsequently stored at −70 °C until DNA extraction.

### 2.7. Histology

Tissue samples were immediately fixed in 10% neutral buffered formalin after excision. After fixation, the samples were rinsed with water and dehydrated using graded alcohols. The tissue was then immersed in xylene for effective clearing and embedded in paraffin wax. Paraffin blocks containing the embedded samples were sectioned to obtain thin slices using a microtome. These tissue sections were transferred onto glass slides. Hematoxylin and eosin staining was performed on the tissue sections. The slides were deparaffinized using xylene and rehydrated through graded alcohols. The sections were then stained with hematoxylin for nuclear staining and eosin for cytoplasmic counterstaining. After staining, the slides were dehydrated, cleared in xylene, and cover-slipped. The stained slides were examined under a light microscope (Olympus), and representative micrographs were captured using a digital camera.

## 3. Results

### 3.1. Index Outbreak

In July 2015, a fish farm in Singapore experienced an outbreak of what later appeared to be a novel viral disease in *L. calcarifer*. Affected fish had generalized skin lesions, became lethargic, and showed pronounced inappetence. As the disease progressed, the skin lesions became more severe, leading to a darkening of the skin with pale whitish patches and erosions of the fins and tail, giving a ghost-like appearance to the fish. Eyes became swollen and slightly cloudy. The internal signs of the disease were enlarged spleen and kidneys. Kidneys became friable and easily detachable. Some pallor of the liver could also be observed. The gills became pale as the disease progressed. The farmer initially thought that the disease was scale drop disease, caused by SDDV. However, diagnostic PCRs for SDDV remained negative and when disease signs were studied more closely, it appeared that the diseased fish showed more peracute clinical signs with a higher morbidity compared to scale drop disease. Disease signs started within 6–10 days post-introduction into sea cages, with high morbidity and up to 60% mortality within 4–7 days from the onset of clinical signs. The skin lesions were also less severe and more generalized compared to scale drop disease, and the whole fish was darker and duller, with patches of pale mucus. Some scale loss was observed, but it was not prominent, nor was it the predominant clinical sign. Scale loss caused by SDDV appears with localized, patchy lesions that are more deeply affected by necrosis with severe scale loss, and it typically causes a more chronic outbreak. The clinical presentation of the fish was not indicative of primary infection with (common) bacterial or parasitic pathogens as the cause, and follow-up investigations on these types of pathogens were therefore not conducted. *T. maritimum* secondary infections were suspected in some clinical cases in the later phase of the disease. Based on the acute presentation and high morbidity, the presence of a yet unidentified viral infectious agent was suspected and further explored.

### 3.2. Virus Culture from Affected Fish

Kidney homogenates (1:9 weight/volume) were established (Section 2.1.3), and subsequently cell-free supernatants obtained by centrifugation were inoculated on cultured sea bass brain cells (SBB cells, MSD Animal Health). A cytopathic effect (CPE) characterized by rounding of the cells followed by detachment and lysis was visible from Day 3 post-inoculation onwards (see Supplementary Figure S2, for a representative picture of the CPE). Three additional passages were performed and in each of the passages the described CPE was visible. A 0.22 µm filter step was applied to the inoculum of Passage 3. The CPE-causing agent from the Singapore farm in July 2015, was subsequently named V511. A representative example of the virus has been deposited with the Collection Nationale de Cultures de Microorganisms (CNCM), Institut Pasteur, 25 Rue du Docteur Roux, F- 75724 Paris Cedex 15, France, under accession number CNCM I-5118.

In January 2016, a new outbreak with similar clinical signs occurred at a different geographical location in Singapore. Again, the SDDV PCR was negative but a PCR developed to detect the by-then partially identified viral agent V511 scored positive on homogenized kidney tissue from affected fish. This CPE-causing agent was subsequently propagated in culture and designated V516.

### 3.3. Virus Identification

VIDISCA-NGS [35], a virus discovery metagenomics test, was performed on the V511 culture supernatant from Passage 1, V511 supernatant from Passage 4, and on pooled serum samples of sick fish of the index outbreak. In all three samples, sequence reads with relatively low but distinguishable similarity to alloherpesviruses were detected, and importantly no other sequence reads with recognizable identity to a virus were found. This suggested that a novel alloherpesvirus, which we named *Lates calcarifer* herpesvirus (LCHV), was present in the serum of sick fish and isolated and cultured on the SBB cells. Amongst the initial reads was a 208 bp fragment that showed homology to the translated nucleotide level with ORF62 of Ictalurid herpes virus 1 NP_041153.2, a terminase gene. Subsequently, PCR primers were designed on this fragment for the initial identification of LCHV V511-positive samples. 

### 3.4. LCHV Genome Characterization and Phylogeny

**Figure 1:**
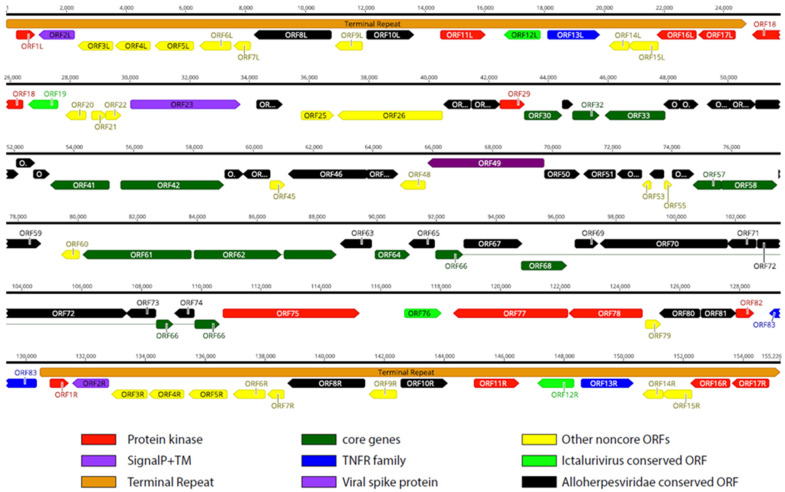
Predicted *Lates calcarifer* herpesvirus (LCHV) isolate V511 genome layout. Predicted functional open reading frames (ORFs) are indicated. The terminal repeats are annotated as an orange arrow box. Conservation degree and gene families are defined in the key at the bottom. Introns are depicted as thin lines connecting the exons (only ORF62 and ORF66).

Illumina NGS and ONT sequencing were performed on the isolates V511 and V516, and the complete genome sequences of the two virus isolates could be obtained (see Supplementary Table S2 for details on ONT sequencing). The complete genome organization of the LCHV isolate V511 is shown in Figure 1 (see Supplementary Figure S3, for the genome layout of LCHV isolate V516). A long unique region of approximately 105 kb is flanked by terminal repeats (TRs) of approximately 24,800 bp. In total, 83 predicted ORFs were found in the LCHV genome, with 17 of these ORFs located in the terminal repeat regions (see Supplementary Table S3 for ORF similarities between the LCHV isolates V511 and V516 and the best-matching functional properties from the closest reference genomes). In amino acid identity searches, 55 of the 83 LCHV putatively encoded proteins showed homology with proteins encoded by members of the genus Ictalurivirus (best-matching were 35 genes of AciHV-2; 13 of IcHV-1; 4 of SiHV-1; and 3 of IcHv-2). Five additional ORFs showed homology to ORFs of other members of the *Alloherpesviridae* family (AngHV-1, LsHV, and CyHV-1). A total of 22 ORFs in LCHV are considered LCHV-specific as no ortholog gene with sufficient homology (>25% at amino acid level) could be identified.

The G+C content of the full LCHV genome was 63.0%. The unique and repeat regions show a slight difference in nucleotide composition, with 60.4% and 68.4% G+C, respectively. The genome sequences of the LCHV isolates V511 and V516 are 89.05% identical at the nucleotide level, and the average identity of the translated ORFs is 89.20%. Although 32 ORFs, 24 ORFs, and 14 ORFs show an amino acid identity of >95%, >90%, and >80% respectively, there are 13 ORFs with a protein identity lower than 80%. The ORFs with the lowest protein identity between the two isolates are: ORF52 (63.3%), ORF21 (62.6%), ORF12L/R (60.6%; L/R indicates positioning in the left or the right TR, respectively), ORF25 (55.6%), and ORF83 (49.4%). The full-length annotated genome sequences of both LCHV isolates were deposited in NCBI GenBank under Bioproject: PRJNA1047000.

We compared the genome of LCHV to the genome of members of the genus *Ictalurivirus*. The genome of LCHV is approximately 21 kb and 5 kb longer than IcHV-1 and SiHV-1, respectively. The length of the TR is equivalent to the TR of SiHV-1, yet the gene structure within the TR differs, with only ORF10L/R of LCHV showing identity to ORF2 of SiHV-1. In contrast, the part of the TR from ORF8L/R until ORF17L/R contains four LCHV ORFs that have homology with IcHV-1 counterparts in the terminal repeat (between 27.1% and 34.0% identity on AA). Of note, the 5′ part of the LCHV TR (8.2 kb) encoding ORF1L/R–ORF7L/R does not show any homology (<25% identity AA) with an ORF of other alloherpesviruses. However, the first ORF (ORF1L/R) showed a 33% homology (AA) with a guanylate kinase protein from a non-*Alloherpesviridae* member, which is the Abalone Herpes Virus (now named Haliotid herpesvirus-1 (HaHV-1, belonging to the genus of *Aurivirus* in the *Malacoherpesviridae* family)). 

**Figure 2:**
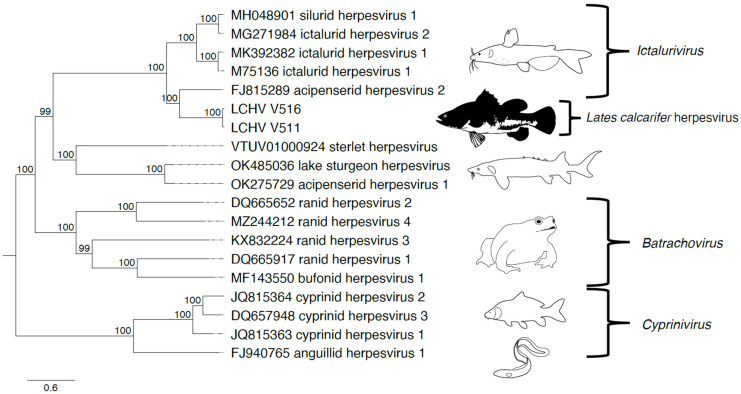
*Alloherpesviridae* phylogeny including *Lates calcarifer* herpesvirus (LCHV). The *Alloherpesviridae* phylogeny inferred using iqtree with the model LG + F + R5 on 12 core genes of 15 reference viruses, 2 references with 1 incomplete gene, and LCHV isolates V511 and V516. On the leaves, the GenBank accession no. and scientific name are displayed. On the right, the genera to which they belong has been indicated. The tree has been midpoint rooted. Bootstrap values are shown at the nodes. Illustrations were adapted from Walker et al. [22].

Phylogeny using the 12 core genes of *Alloherpesviridae* shows that LCHV clusters with members of the genus *Ictalurivirus* (Figure 2, Supplementary Table S4). 

### 3.5. Association with Disease

To study the association with disease in sampling sets collected from the field (Section 2.1.1), diagnostic PCR tests were performed (Section 2.4.2). A diagnostic sampling set was scored positive if at least one of the submitted pool samples tested positive in the LCHV diagnostic nested PCR. In total, 86 pooled organ or serum samples of healthy or suspected diseased fish were tested, and 39 of these were PCR positive for the LCHV virus genome. The sampling sets from one farm in Indonesia (2014) and one farm in Singapore (sampled at four different time points in 2014–2015 prior to the first reports of the novel virus) tested negative for LCHV. Of note, two sampling sets from the Singapore farm tested positive for SDDV, while not showing clinical disease. Of the six farms suspected of LCHV infection, five tested positive in PCR: three out of three farms in Singapore (6/8 samplings), one out of one farm in Vietnam (2/2 samplings), one out of one farm in Sri Lanka (2/2 samplings), and zero out of one farm in Saudi Arabia (1 sampling). Of note, one of the LCHV positive farms in Singapore also showed a concomitant SDDV infection. A set of pooled serum samples from healthy brood stock fish at MSD Animal Health served as a negative control and tested negative as well.

### 3.6. Experimental Infection of LCHV V511 in L. calcarifer and Reproduction of the Clinical Signs

To prove that LCHV was the cause of the disease observed, we carried out intra-peritoneal injection of cell-free cultured LCHV V511 virus (Passage 4), setting up a series of experimental infection studies in naïve fish. The pilot experiment comprised a dose finding setup (3.1 × 10^5^, undiluted or 3.1 × 10^4^ TCID_50_/fish, 10× diluted) with concomitant investigation if cohabitant fish separated by netting from experimentally infected fish showed signs of infection. The first signs of disease, loss of appetite, skin and fin lesions, and lethargy appeared at Day 3 in the IP groups (undiluted and diluted), and at Day 7 in the cohabitant groups (diluted and undiluted). On Day 9, food intake had ceased, a loss of swimming equilibrium was observed, and gaping started with an increase in respiratory rate, in both infected tanks (see Supplementary Video S1). Darkening of the skin became apparent on Day 14 and swelling of the eyes on Day 15 (see Supplementary Videos S2 and S3) in both the IP and cohabitant groups (diluted and undiluted). Mortalities were limited to one cohabitant fish in the undiluted 3.1 × 10^5^ dose tank at Day 14 when the study ended at Day 21 post-infection. 

**Figure 3:**
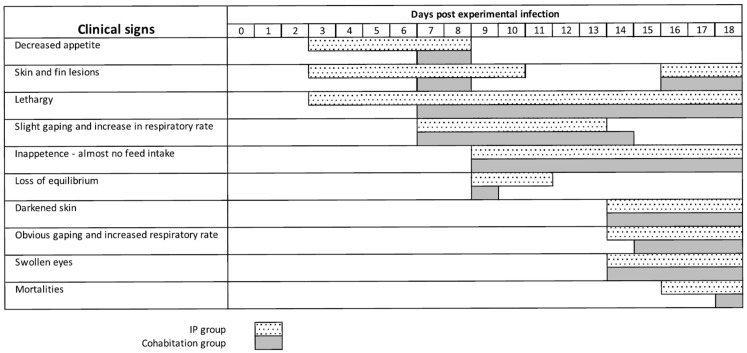
Clinical signs after the first main infection experiment and natural infection in the cohabitant group.

In the first main infection experiment, where we used a 9.3 × 10^5^ TCID_50_ dose per fish, 25 infected fish were cohabitated with 25 uninfected fish in the same tank, separated by netting. The clinical signs of both groups were monitored each day and summarized in Figure 3. The experimentally infected fish and the cohabitant fish showed grossly the same clinical signs of disease, which resembled the signs observed in the fish from which the virus was cultured. The first signs of disease, loss of appetite, skin and fin lesions, and lethargy appeared at Day 3 in the IP group, and at Day 7 in the cohabitant group. On Day 9, food intake had ceased, a loss of swimming equilibrium was observed, and gaping started with an increase in respiratory rate, in both groups (see Supplementary Video S1). Darkening of the skin became apparent on Day 14 and swelling of the eyes on Day 15 (see Supplementary Videos S2 and S3). Mortality was seen in the IP group on Day 16 (8%), Day 17 (69%, cumulative mortality), and Day 18 (77%, cumulative mortality) (see Supplementary Figure S4A). In the cohabitant fish, mortality started one day later, but reached the same cumulative mortality by Day 18 (77%). It was decided to terminate the experiment and bleed the remaining fish because most likely not enough fish would have survived for the collection of materials at Day 21.

### 3.7. Confirmation of LCHV Infection in Experimentally Infected Fish/Cohabitant Fish

During the first main infection experiment, 3 fish per group were randomly sampled for collection of organs at Day 4, 7, 11, 14, and 17/18 post-infection (dpi). The final sampling point for the experimentally infected fish was Day 17, and Day 18 for the cohabitant fish. At Day 17/18 post-challenge, all fish were either sampled or had succumbed to the infection. Detailed dissemination data were obtained from the cohabitant fish days on Days 4–7–11–14–18 and from experimentally infected fish on Day 17. In addition to that, kidneys were sampled on Days 4–7–11–14 from the experimentally infected fish. A relatively high LCHV DNA copy number was detected in kidneys from experimentally infected fish at 4 days post-infection. Viral genome counts in this organ decreased over time to about 200 copies per mg kidney tissue on Day 17 (Table 1). On this day, high quantities of LCHV genomes were detected in the other organs. The highest LCHV genome copy numbers were found in the gills, skin, heart, and serum (Table 1).

In cohabitation-infected fish, LCHV was detected in all organs except for the spleen and serum at Day 4 (Table 1). The highest copy numbers of the virus were detected in skin. Between Day 4 and Day 14, a clear decrease in virus DNA copy numbers was seen in all organs, and LCHV DNA was detected in only four organs on Day 7. On Days 11 and 14, LCHV DNA could only be detected in skin and brain samples. On Day 18, around peak mortality, LCHV reappeared massively in all organs except the spleen. The highest copy numbers of the virus were present in the gills, heart, and skin. Overall, the qPCR data in the cohabitant group showed a biphasic viremia with peaks shortly after infection and with the onset of clinical signs and mortality. Of note is the absence of DNA in the serum from cohabitant fish, except for on Day 18. In conclusion, we showed with these experiments that the LCHV isolate V511 infects Barramundi, replicates, disseminates to different organs, and causes clinical disease with variable mortalities.

A second main infection experiment was set up to collect organs for histopathological analysis (see Section 3.9, IP-infected fish only) and to collect materials for re-isolation of the virus in SBB cell culture after infection. We initially cultured the virus from kidney homogenates collected around peak clinical signs on a farm in the field, but at the time it was unclear which sample material would best qualify for re-isolation of the virus. In the second main IP infection experiment, the same clinical signs were observed in heavier fish, with a somewhat different presentation in time (see Supplementary Figure S5). The mortality in this experiment was lower and the study could be completed as planned on Day 21, with a cumulative mortality of 27% in the cohabitant group (see Supplementary Figure S4B).

### 3.8. Electron Microscopy Visualization of LCHV

**Figure 4:**
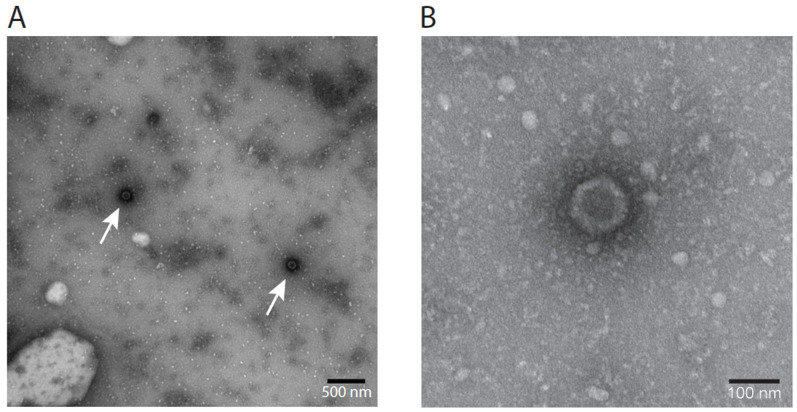
Electron microscopy of cultured LCHV V511. (**A**) Non-enveloped icosahedral structures characteristic of herpesviruses were detected by EM in cultured LCHV, isolate V511, indicated by arrows. (**B**) Five times magnification of the structure indicated by the left arrow in panel A.

Herpesviruses carry a dense core in an icosahedral capsid (100 nm in size), surrounded by an envelope [49]. In between the envelope and the capsid is the proteinaceous tegument. In negative-stain EM, various presentations of the virus can be encountered. As intact envelopes are impermeable to negative stains, intact viruses are not visible by uranyl acetate staining, yet virions with damaged envelopes are visible in EM, either appearing like sunny side up eggs with diameters larger than the original viruses, or as only icosahedral capsids of 100 nm with a dense core, lacking the envelope. In virus harvest (Passage 5), LCHV capsid structures were visible as dark spots with an approximate diameter of 100 nm, matching the average diameter of the capsid of common herpes viruses (115–130 nm, Figure 4A). A five times magnification (Figure 4B) clearly shows the icosahedral contour of an unenveloped virus particle. 

### 3.9. Histopathological Analysis of Experimentally Infected Fish

**Figure 5:**
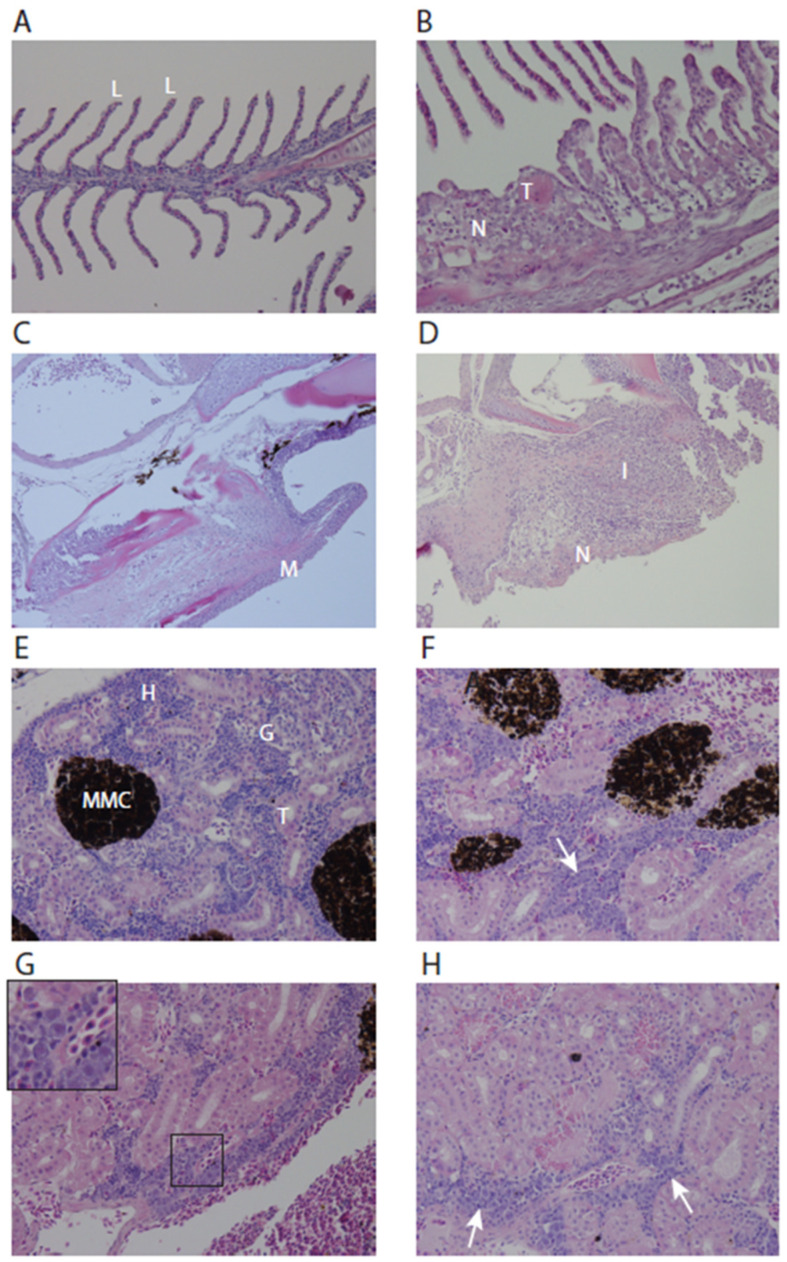
Gill and kidney histopathology of control and experimentally LCHV-infected fish. (**A**) Normal gill filament with details of lamellae “L”, 400× magnification; (**B**) Gill filament of LCHV experimentally infected fish at 17 dpi. Lamellae show shortening, blunting, adhesion, epithelial hypertrophy/hyperplasia, scattered necrosis “N”, and microthrombi “T”, 400× magnification. (**C**) Normal gill arch, “M” in the picture indicates mucosa, 200× magnification. (**D**) Gill arch of LCHV experimentally infected fish at 17 dpi. Detail of the mucosal necrosis “N” and inflammation “I”, magnification 200×. (**E**) Normal kidney, “H” = hematopoietic tissue, “MMC” = melanomacrophage center, “T” = tubules, “G” = glomeruli, 400× magnification. (**F**) Kidney LCHV experimentally infected fish at 14 dpi. Note that in the hematopoietic tissue, a larger proportion consists of large blastoid cells (arrow), 400× magnification. (**G**) Kidney at 14 dpi with suspect intranuclear inclusions in blastoid cells (inset), 400× magnification. (**H**) Kidney at 17 dpi. Intracytoplasmic, eosinophilic hyaline droplets in the tubular epithelium and presence of blastoid cells (arrows), 400× magnification. All panels: H&E stain.

Histopathologic lesions in the gills and kidney of experimentally infected fish related to the viral infection were examined at 14 days and 17 days post-infection in the second main infection experiment. The lesions in the gills of infected fish reflected a fairly non-specific reaction pattern, with lamellar abnormalities (Figure 5A,B) and necrosis of lamellar pavement cells and the mucosa lining of the gill arch (Figure 5C,D). No histological abnormalities were observed in the brain, liver, spleen, and heart. Corneal ulceration was observed in two submitted eyes (not shown). In the kidneys, hyperplasia of the hematopoietic tissue with suspect intranuclear inclusions was visible at 14 dpi (Figure 5E–H). The hyperplasia of hematopoietic tissue can be a compensatory response to systemic inflammation. It is of note that this observation may be related to the high viremia in the kidneys of experimentally infected fish and may not have been present in cohabitant fish (see Table 1). 

## 4. Discussion

We identified and characterized a novel virus causing a distinct clinical disease in *L. calcarifer* and fulfilled Koch’s postulates for causality. Although Koch’s postulates [50] were originally not defined for viral diseases because they were unknown at the time, we completed the steps of virus identification and virus isolation from diseased fish, experimental infection, reproduction of clinical signs, re-isolation of the novel infectious agent LCHV from the diseased fish, and thus fulfilled the postulates. LCHV is the first herpesvirus described that is pathogenic to Barramundi. The biology of the virus shares similarities with other herpesviruses in fish such as an epitheliotropic nature and a biphasic viremia. Based on phylogeny, the Ictalurid herpesvirus 1 (IcHV-1) and Acipenserid herpesvirus 2 (AciHV-2) are the closest family members of LCHV. IcHV-1 is pathogenic for channel catfish, and amongst the signs are disorientation during swimming and bulging eyes, which were also observed in experimental LCHV infection. IcHV-1 also causes damage to the skin. For AciHV-2, skin ulceration, lethargy, inappetence, and erratic swimming have been reported as clinical signs. LCHV, IcHV-1, and AciHV-2 thus share at least some clinical signs [51].

We describe the full-length genome sequences of two isolates of LCHV. The two isolates, collected at different farms, can be regarded as two distinguishable variants of the same virus. A genome identity of 89% was found, and the identity of the putative proteins encoded by the viruses show a similar identity. However, there appear to be notable differences between the isolates V511 and V516, the most remarkable difference being the variation in the ORF83 sequence, with only 49.6% amino acid identity. More exactly, this ORF contains a TNF-receptor family domain, which is usually involved in immune evasion. Whether this variation may result in an increased or decreased morbidity and mortality is uncertain, as we have only performed experimental infections with one of the two isolates (V511). The V516 isolate was obtained from fish which presented with similar clinical signs as the fish that were infected by the isolate V511. 

The genome of LCHV contains unique features. The first 8.2 kb of terminal repeats do not show any similarity to genome parts of other known members within the *Alloherpesviridae* family. Strikingly, this region specifically contains an ORF with a 33.1% match (AA) to a putative guanylate kinase gene in Haliotid herpesvirus-1 (HaHV-1; previously called abalone herpesvirus) [52]. This virus is the causative agent of acute ganglioneuritis in abalone or *Haliotis* spp. which are edible gastropod mollusks [53]. The function of guanylate kinase is to catalyze the transfer of a phosphate group from ATP (adenosine triphosphate) to GMP (guanosine monophosphate), leading to the formation of GDP (guanosine diphosphate). This contributes to the control of nucleotide pool concentrations, as this enzyme is involved in purine metabolism [54]. We also noted that the isolates V511 and V516, collected in Singapore within one year from each other, show considerable genetic variation (>10% at nucleotide level), suggesting that the virus variants have evolved separately for multiple generations and are only distantly related.

Although the disease signs caused by LCHV may be clearly distinguishable in experimental conditions, they may be hard to distinguish under field conditions. During the last two decades, diseases in the maricultures of *L. calcarifer* have gained more attention, which has led to the identification of novel viruses and their associated pathology and disease signs. Signs caused by different viral pathogens are now known to overlap, for example the scale loss caused by LCHV has much in common with the signs of scale drop disease, but through increased knowledge building and diagnostics, diseases can now be better identified. Furthermore, viral diseases of the seabass skin are often accompanied by bacterial infections such as *T. maritimum* [55]. Of note, LCHV disease is more acute than SDDV disease, the inappetence is pronounced, and lethargy is one of the first signs, followed by disorientated swimming. SDDV-infected fish will continue to consume food. Despite these distinct clinical characteristics, it is possible and likely that LCHV circulated long before 2015 but has remained unnoticed for years. 

Since we first described the virus [9], the virus has also been described by Meemetta et al., (2020) [10]; Domingos et al., 2021 [11]; and Dang et al., 2023 [12]. Also, two sequences were submitted to GenBank (LC438644 and LC438645), indicating that the virus was also present in Vietnam in 2015 (see Supplementary Figure S6). We realize that our pathohistological analysis of LCHV is not fully congruent with published work. Dang et al. (2023) [12] performed a detailed histological examination of experimentally infected Barramundi, albeit that the inoculum used for the study was not pure (not passaged over cell culture) and contained a different variant of LCHV. These authors reported the presence of inclusion bodies in multiple organs, including the liver, pancreas, kidney, eyes, gills, and adipose tissue. They state that the presence of these inclusion bodies can be used particularly for the surveillance of this viral disease. To some extent, inclusions were also observed in our study in the kidney at Day 14 post-infection, and the inclusions grossly look alike. However, diagnosis based on inclusion bodies needs confirmation by ultrastructural analysis. Importantly, our fish suffered from severe disease, yet strong histopathology in most organs was not found, therefore inclusion body occurrence alone may not always be a reliable histological marker for LCHV infection. Of note, the histopathological abnormalities in the liver observed in our studies may be related to the iatrogenic route for infection.

Our study setup with cohabitant fish provides insight in the initial dissemination, as well as replication, of the virus under natural conditions, although the exact sites of replication have not been elucidated. Initial replication likely occurs in the gills based on our findings in the cohabitant fish. The lethargy and disorientated swimming that precede the phase of severe clinical signs can be related to the moderate but constant viral load in the brain, although at present it is not known where in the brain the virus resides. No overt histological abnormalities were observed in the brain. From Day 14 onwards, an increase in viral load was found in various organs, although histological abnormalities were limited to gills and maybe the kidney. It is not clear from our study whether gill failure or a generalized organ failure causes mortality. 

We have not investigated whether latency occurs. Quite possibly, fish that survive an infection may become latently infected, a feature which is the hallmark of all herpesviruses. For CyHV1, CyHV3, SalHV2, and IcHV1, latency has indeed been studied (reviewed by Hanson et al. [13]) and experimentally demonstrated for CyHV3 [15]. Whether latency occurs, and if so, whether latently LCHV infected fish can at a later stage start shedding infectious LCHV particles remains to be determined. 

In conclusion, we identified and genetically characterized *Lates calcarifer* herpesvirus, a novel herpesvirus causing significant losses in commercial fish populations. The complete genome sequence revealed a combination of regions with homology to known fish herpesviruses, as well as unique genetic elements, especially in the TRs, which have not been described in these viruses previously. Pathohistological analysis showed marked alterations in infected gills, but further studies are required to elucidate the exact biology of this large DNA virus.

## Figures and Tables

**Table 1:** LCHV DNA copy number concentrations in tissue and serum samples after experimental or cohabitant infection (first main infection experiment).

	Days Post-Challenge				
	4	7	11	14	17 or 18 ^a^
Intraperitoneal injection					
Gills	0 ^b^	nt	nt	nt	36,214
Heart	nt	nt	nt	nt	5154
Intestine	nt	nt	nt	nt	3481
Brain	nt	nt	nt	nt	582
Liver	nt	nt	nt	nt	1628
Skin	nt	nt	nt	nt	39,032
Spleen	nt	nt	nt	nt	nt
Kidney	30,167	19,204	262	46	214
Serum	nt	nt	nt	nt	21,056
Cohabitant infection					
Gills	403	80	0	0	15,060
Heart	533	31	0	0	2930
Intestine	2731	7	0	0	29
Brain	234	65	20	50	25
Liver	836	0	0	0	162
Skin	748,800	7	7	831	16,111
Spleen	nt	0	0	0	nt
Kidney	12	85	0	0	6
Serum	0	0	0	0	730

^a^: Day 17 for the intraperitoneal (IP) injected fish, and Day 18 for the cohabitant infection; ^b^: DNA copies per mg (tissues) or per mL (serum); nt: not tested (no samples taken). All organ and serum samples contained pooled materials from 3 fish. On Day 17/18, samples from all remaining fish were pooled.

## Data Availability

Data generated in this project are available at NCBI under Bioproject Code PRJNA1047000.

## Supplementary Materials

The following supporting information can be downloaded at: https://www.mdpi.com/article/10.3390/genes15030264/s1, Supplementary Table S1: Twenty reference genomes used for ORF prediction and annotation of the LCHV genomes; Supplementary Table S2: Oxford Nanopore Technologies sequencing data overview; Supplementary Table S3: ORF similarities between the LCHV strains V511 and V516 and the best-matching functional properties from closest reference genomes; Supplementary Table S4: Sequences of 12 Alloherpesviridae core genes for phylogenetic analysis; Supplementary Figure S1: Overview of oligonucleotides used for the detection of LCHV ORF66; Supplementary Figure S2: Microscopy pictures showing SBB cell morphology and CPE caused by LCHV infection; Supplementary Figure S3: Genome map of LCHV isolate V516; Supplementary Figure S4: Graph showing cumulative mortality in the two main LCHV infection experiments; Supplementary Figure S5: Clinical signs after the second main infection experiment and natural infection in the cohabitant group of the second main experiment; Supplementary Figure S6: Phylogenetic analysis of 22 alloherpesviruses and three sequences of LCHV isolates; Supplementary Video S1: LCHV-IP-infected fish show spiral swimming at Day 10 post-infection. Notice that fish also show prominent scale loss. Food is visible at the bottom of the tank, indicating that LCHV-infected fish completely lost their appetite. This is in sharp contrast to SDDV-infected fish, which would still eat normally; Supplementary Video S2: LCHV-IP-infected fish at Day 15 post-infection. Fish are lethargic and show obvious gaping; Supplementary Video S3: LCHV-Cohab fish at Day 15 post-infection. Large quantities of food are visible at the bottom of the tank due to inappetence. Fish are lethargic and show obvious gaping. Fish show significant muscle mass loss.
