# Supplementary material for: Discovery, Pathogenesis, and Complete Genome Characterization of Lates calcarifer Herpesvirus
Source: Genes (Basel). 2024 Feb 20;15(3):264. doi: 10.3390/genes15030264 (PMC10970581; doi:10.3390/genes15030264)
Supplement: Supplementary file 1 [file genes-15-00264-s001.zip › Supplementary_Material.pdf]

Supplementary material
Discovery, pathogenesis, and complete genome characterization of Lates Calcarifer herpesvirus

Bartjan Simmelink [1], Jordy P.M. Coolen [2], Wannes Vogels [1], Martin Deijs [3,4], Jessica L.M. van der Last-Kempkes [1], Kah Sing Ng [5], Siow Foong Chang [6], Koen Gevers [1], Liesbeth Harkema [7] , Lia van der Hoek [3,4], Ad de Groof [1,*]

[1] Department Discovery & Technology, MSD Animal Health, Wim de Körverstraat 35, P.O. Box 31, 5830 AA Boxmeer, The Netherlands; bartjan.simmelink@merck.com; wannes.vogels1@merck.com; jessica.van.der.last@merck.com; koen.gevers@merck.com; ad.de.groof@merck.com
[2] Department R&D-IT, MSD Animal Health, Wim de Körverstraat 35, P.O. Box 31, 5830 AA Boxmeer, The Netherlands; jordy.coolen@merck.com
[3] Amsterdam UMC, Laboratory of Experimental Virology, Department of Medical Microbiology and Infection Prevention, University of Amsterdam, Meibergdreef 9, 1105 AZ, Amsterdam, The Netherlands; m.deijs@amsterdamumc.nl; c.m.vanderhoek@amsterdamumc.nl
[4] Amsterdam Institute for Infection and Immunity, Postbus 22660, 1100 DD, Amsterdam, The Netherlands
[5] MSD Animal Health Innovation Pte Ltd, 1 Perahu Road 718847 Singapore; kah.sing.ng@msd.com
[6] MSD Animal Health Innovation Pte Ltd , present address Animal & Veterinary Service, National Parks Board, 1 Cluny Road, Singapore Botanic Gardens, 259569 Singapore; chang_siow_foong@nparks.gov.sg
[7] MSD Animal Health Pathology, present address Laboratory for Pathology and Histology, Royal GD, Arnsbergstraat 7, 7418 EZ Deventer, the Netherlands; l.harkema@gddiergezondheid.nl
* Correspondence: ad.de.groof@merck.com

Content:

- **Supplementary Table S1.** Twenty reference genomes used for ORF prediction and annotation of the LCHV genomes.

- **Supplementary Table S2.** Oxford Nanopore Technologies sequencing data overview.

- **Supplementary Table S3**. ORF similarities between LCHV strains V511, V516 and best matching functional properties from closest reference genomes.

- **Supplementary Table S4.** Sequences of 12 Alloherpesviridae core genes for phylogenetic analysis.

- **Supplementary Figure S1.** Overview of used oligonucleotides for detection of LCHV ORF66.

- **Supplementary Figure S2.** Microscopy pictures showing SBB cell morphology and CPE caused by LCHV infection.

- **Supplementary Figure S3.** Genome map of LCHV isolate V516.

- **Supplementary Figure S4.** Graph showing cumulative mortality in the two main LCHV infection experiments.

- **Supplementary Figure S5.** Clinical signs after second main infection experiment, and natural infection in the cohabitant group of the second main experiment.

- **Supplementary Figure S6.** Phylogenetic analysis of 22 alloherpesviruses and three sequences of LCHV isolates.

- **Supplementary Movie S1.** LCHV-IP-infected fish show spiral swimming at day 10 post infection. Notice that fish also show prominent scale loss. Food is visible at the bottom of the tank, indicating that LCHV-infected fish completely lost their appetite. This is in sharp contrast to SDDV infected fish, which would still eat normally.

- **Supplementary Movie S2.** LCHV-IP-infected fish at day 15 post infection. Fish are lethargic and show obvious gaping.

- **Supplementary Movie S3.** LCHV-Cohab fish at day 15 post infection. Large quantities of food are visible at the bottom of the tank due to in appetence. Fish are lethargic and show obvious gaping. Fish showed significant muscle mass loss.

Supplementary Table S1.

Name	Description	Organism	Virus abreviation
KU096999.1	Abalone herpesvirus Taiwan_2005, partial genome	Abalone herpesvirus	AbaHV-Taiwan
NC_018874.1	Abalone herpesvirus Victoria_AUS_2009, complete genome	Abalone herpesvirus	AbaHV-Victoria
OR001786.1	Acipenserid herpesvirus 1 strain UC Davis, partial genome	Acipenserid herpesvirus	AciHV-1
FJ815289.2	Acipenserid herpesvirus 2 strain SRWSHV, partial genome	Acipenserid herpesvirus 2	AciHV-2
FJ940765.3	Anguillid herpesvirus 1 strain 500138, complete genome	Anguillid herpesvirus	AngHV-1
MF143550.1	Bufonid herpesvirus 1 strain FO1_2015, complete genome	Bufonid herpesvirus	BufonidHV-1
GQ153938.1	Chlamys acute necrobiotic virus, complete genome	Chlamys acute necrobiotic virus	AVNV
JQ815363.1	Cyprinid herpesvirus 1 strain NG-J1, complete genome	Cyprinid herpesvirus	CyHV-1
JQ815364.1	Cyprinid herpesvirus 2 strain ST-J1, complete genome	Cyprinid herpesvirus	CyHV-2
DQ657948.1	Cyprinid herpesvirus 3 strain KHV-U, complete genome	Cyprinid herpesvirus	CyHV-3
MW412419.1	Haliotid herpesvirus 1 isolate CN2003, complete genome	Haliotid herpesvirus	HalioHV-1
M75136.2	Ictalurid herpesvirus 1 strain Auburn 1, complete genome	Ictalurid herpesvirus	IcHV-1
MK392382.1	Ictalurid herpesvirus 1 strain S98-675, complete genome	Ictalurid herpesvirus	IcHV-1
MG271984.1	Ictalurid herpesvirus 2 strain 760_94, complete genome	Ictalurid herpesvirus	IcHV-2
OK485036.1	Lake sturgeon herpesvirus strain Wolf River, complete genome	Lake sturgeon herpesvirus	LsHV
OQ101585.1	Ostreid herpesvirus 1 isolate SDYK2017, complete genome	Ostreid herpesvirus	OsHV-1
DQ665917.1	Ranid herpesvirus 1 strain McKinnell, complete genome	Ranid herpesvirus	RaHV-1
DQ665652.1	Ranid herpesvirus 2 strain ATCC VR-568, complete genome	Ranid herpesvirus	RaHV-2
KX832224.1	Ranid herpesvirus 3 isolate FO1_2015, complete genome	Ranid herpesvirus	RaHV-3
MH048901.1	Silurid herpesvirus 1 strain KRB14001, complete genome	Silurid herpesvirus	SiHV-1

Supplementary Table S2.

	ONT reads	Assembly [a]			Final assembly [b]			
	Number of reads	Mean read length	Mean read quality	Assembly size	Mean coverage	Total Genome size	Unique region	Repeat region
**LCHV V511**	543,497	1,634	14.4	130,479	37 x	155,226	105,742	24,742
**LCHV V516**	1,530,296	2,351	14.2	129,498	19 x	154,368	104,684	24,842

[a] The initial assembly resulted in a circular genome of 130,479 and 129,498 for V511 and V516, respectively.

[b] Genome termini as well as Terminal Repeat boundaries were determined by visual inspection and read depth distribution based on mapped Illumina sequencing reads.: **V511**: 2,071,650 of 2,698,070 Illumina reads (76.8%); **V516**: 163,459 of the 878,994 (18.6%) Illumina reads. After manual curation of homopolymer sequences based on Illumina sequencing reads the final assembly was obtained.

Supplementary Table S3.

LCHV strain V511	LCHV strain V516			Best match					Protein domains, properties and / or putative function
**ORF**	**ORF**	**Identity AA (%)[a]**	**Alignment length**	**Organism[b]**	**Common name**	**Accession number**	**Identity AA (%)**	**alignment length**	
ORF1L/R	ORF1L/R	94.4	197	AbaHV	guanylate kinase	YP_006908763.1	33.1	181	PF00625 Guanylate kinase
ORF2L/R	ORF2L/R	67.7	403						Signal peptide VVG-EL probability 0.8306
ORF3L/R	ORF3L/R	97.9	389						
ORF4L/R	ORF4L/R	91.0	388						
ORF5L/R	ORF5L/R	90.9	429						
ORF6L/R	ORF6L/R	94.3	352						IPR011029 Death-like domain superfamily
ORF7L/R	ORF7L/R	93.7	159						
ORF8L/R	ORF8L/R	84.3	870	IcHV-1	ORF1L	QAB08485.1	27.1	554	
ORF9L/R	ORF9L/R	92.7	303						
ORF10L/R	ORF10L/R	85.9	539	SiHV-1	ORF2	AVP72179.1	25.4	276	
ORF11L/R	ORF11L/R	95.5	494	IcHV-1	ORF5	AAA88108.1	34.0	191	PF01712 Deoxynucleoside kinase
ORF12L/R	ORF12L/R	60.6	401	AngHV-1	ORF66	ADA57829.1	29.8	235	Signal peptide SDG-AP probability 0.6567
ORF13L/R	ORF13L/R	77.6	595	AngHV-1	ORF65	ADA57828.2	31.5	330	PF01823 Membrane Attack Complex (MAC) /Perforin domain
ORF14L/R	ORF14L/R	96.1	205						
ORF15L/R	ORF15L/R	83.3	330						TMHMM - AA 37-59
ORF16L/R	ORF16L/R	94.5	416	IcHV-1	ORF14	AAA88117.1	31.9	360	PF00069 Protein kinase domain
ORF17L/R	ORF17L/R	99.0	409	IcHV-1	ORF14	AAA88117.1	27.7	383	PF00069 Protein kinase domain
ORF18	ORF18	91.9	492	IcHV-1	ORF16	AAA88119.1	25.6	360	IPR000719 Protein kinase domain
ORF19	ORF19	86.1	274	LsHV	ORF125	UMM52803.1	36.0	100	PF02393 US22 like
ORF20	ORF20	76.7	210						
ORF21	ORF21	62.6	163						
ORF22	ORF22	90.1	172						
ORF23	ORF23	88.0	1219						IPR010916 TonB box, conserved site | Signal peptide ARG-DV probability 0.9698
ORF24	ORF24	97.2	287	SiHV-1	ORF19	AVP72196.1	28.2	131	Signal peptide ALA-AP probability 0.9462
ORF25	ORF25	55.6	365						

ORF26	ORF26	78.7	1163						PTHR32083 Cilia- and flagella-associated protein 58 - AA 437-917
ORF27	ORF27	92.7	300	SiHV-1	ORF23	AVP72200.1	25.8	295	
ORF28	ORF28	98.4	310	AciHV-2	ORF24	AEF97682.1	45.9	242	
ORF29	ORF29	98.5	268	AciHV-2	ORF73	AEF97683.1	35.8	193	PF00069 Protein kinase domain
ORF30	ORF30	98.3	416	IcHV-2	ORF25	AUG72341.1	41.1	496	PF13245 AAA domain | Alloherpesvirus core gene : Helicase
ORF31	ORF31	90.4	114	AciHV-2	ORF26	AEF97685.1	37.5	96	
ORF32	ORF32	94.9	294	AciHV-2	ORF27	AEF97686.1	50.5	289	Alloherpesvirus core gene : capsid triplex protein 2
ORF33	ORF33	91.5	599	IcHV-1	ORF28	QAB08513.1	36.3	612	Alloherpesvirus core gene : capsid maturational protease
ORF34	ORF34	90.4	177	AciHV-2	ORF29	AEF97688.1	44.6	168	
ORF35	ORF35	98.5	205	AciHV-2	ORF30	AEF97689.1	55.6	214	
ORF36	ORF36	90.3	248	AciHV-2	ORF80	AEF97690.1	34.9	186	
ORF37	ORF37	95.5	288	AciHV-2	ORF33	AEF97691.1	46.0	287	
ORF38	ORF38	97.5	358	AciHV-2	ORF34	AEF97692.1	55.0	358	
ORF39	ORF39	96.6	205	AciHV-2	ORF35	AEF97693.1	34.9	195	
ORF40	ORF40	83.2	173	AciHV-2	ORF36	AEF97694.1	35.6	104	
ORF41	ORF41	95.1	652	AciHV-2	ORF37	AEF97695.1	47.9	591	Alloherpesvirus core gene : Allo37 protein
ORF42	ORF42	98.2	1143	AciHV-2	ORF39	AEF97697.1	53.8	1196	Alloherpesvirus core gene : major capsid protein
ORF43	ORF43	99.5	205	AciHV-2	ORF81	AEF97698.1	54.7	201	
ORF44	ORF44	97.0	296	AciHV-2	ORF41	AEF97699.1	39.6	298	
ORF45	ORF45	71.6	169						
ORF46	ORF46	95.4	873	AciHV-2	ORF43	AEF97701.1	50.5	889	
ORF47	ORF47	91.5	342	IcHV-1	ORF44	QAB08528.1	46.9	335	
ORF48	ORF48	75.4	284						
ORF49	ORF49	76.8	1298	AciHV-2	ORF46	AEF97704.1	39.3	1247	SSF58069 Virus ectodomain - AA 879-953 SSF51445 (Trans)glycosidases
ORF50	ORF50	80.8	391	AciHV-2	ORF47	AEF97705.1	40.6	355	PF00082 Peptidase S8 - AA 74-254
ORF51	ORF51	83.4	356	AciHV-2	ORF48	AEF97706.1	41.5	130	
ORF52	ORF52	63.3	275	AciHV-2	ORF49	AEF97708.1	49.3	140	PF00692 dUTPase
ORF53	ORF53	72.3	101						TMHMM - AA 13-35
ORF54	ORF54	80.8	151	AciHV-2	ORF86	AEF97710.1	30.2	116	TMHMM - AA 33-55 | TMHMM - AA 84-103

									| TMHMM - AA 113-135
ORF55	ORF55	87.3	79						
ORF56	ORF56	90.9	242	AciHV-2	ORF52	AEF97712.1	32.2	211	
ORF57	ORF57	96.2	312	AciHV-2	ORF53	AEF97713.1	44.6	316	capsid triplex protein 1
ORF58	ORF58	92.6	623	AciHV-2	ORF54	AEF97714.1	46.0	626	Alloherpesvirus core gene : Allo54 protein
ORF59	ORF59	88.6	396	AciHV-2	ORF55	AEF97715.1	38.8	394	
ORF60	ORF60	84.9	199						
ORF61	ORF61	98.7	1202	AciHV-2	ORF56	AEF97716.1	53.6	1206	Alloherpesvirus core gene : Allo56 protein
ORF62	ORF62	98.6	1548	AciHV-2	ORF57+ORF58	ACZ55868.2	57.3	1549	PF03104 and PF00136 | Alloherpesvirus core gene : DNA polymerase
ORF63	ORF63	93.4	348	AciHV-2	ORF59	ACZ55869.2	26.5	339	major envelope protein | TMHMM - AA 47-69 | TMHMM - AA 101-123 | TMHMM - AA 144-166 | TMHMM - AA 266-288
ORF64	ORF64	97.9	384	IcHV-2	ORF60	ACZ55875.1	38.6	347	Alloherpesvirus core gene : Allo60 protein
ORF65	ORF65	97.2	287	AciHV-2	ORF61	ACZ55871.1	37.8	270	
ORF66	ORF66	98.6	759	IcHV-1	ORF62	QAB08546.1	62.5	723	IPR027417 P-loop containing nucleoside triphosphate hydrolase Alloherpesvirus core gene : DNA packaging terminase subunit 1
ORF67	ORF67	91.3	647	AciHV-2	ORF63	AEF97717.1	44.0	655	Alloherpesvirus core gene : Primase
ORF68	ORF68	96.0	505	AciHV-2	ORF64	AEF97718.1	47.3	509	Alloherpesvirus core gene : Allo64 protein
ORF69	ORF69	86.5	252	IcHV-1	ORF79	QAB08561.1	31.0	87	SSF57924 Inhibitor of apoptosis (IAP) repeat
ORF70	ORF70	80.2	1434	AciHV-2	ORF65	AEF97719.1	33.2	757	IPR038765 Papain-like cysteine peptidase superfamily
ORF71	ORF71	90.6	342	AciHV-2	ORF66	AEF97720.1	42.6	258	SSF81995 beta-sandwich domain of Sec23/24
ORF72	ORF72	98.6	1594	AciHV-2	ORF67	AEF97721.1	37.0	1572	
ORF73	ORF73	95.7	326	AciHV-2	ORF68	AEF97722.1	42.8	320	

ORF74	ORF74	93.6	220	IcHV-1	ORF70	AAA88173.1	44.0	218	
ORF75	ORF75	92.9	1529	IcHV-1	ORF72	AAA88174.1	39.6	442	IPR011009 Protein kinase-like domain superfamily | tegument-associated protein
ORF76	ORF76	92.3	404	CyHV-1	ORF41	AFJ20342.1	31.7	60	PF13920 - Zinc finger, C3HC4 type (RING finger)
ORF77	ORF77	93.2	1273	IcHV-1	ORF73	QAB08555.1	34.2	728	PF00069 Protein kinase domain
ORF78	ORF78	99.6	818	IcHV-1	ORF73	AAA88175.1	33.1	541	PF00069 Protein kinase domain | tegument-associated protein
ORF79	ORF79	98.8	170						
ORF80	ORF80	96.9	447	SiHV-1	ORF75	AVP72246.1	27.8	392	
ORF81	ORF81	96.7	392	IcHV-2	ORF78	AUG72324.1	38.6	365	
ORF82	ORF82	95.5	198						PFAM02224 Cytidylate kinase
ORF83	ORF83	49.4	443	LsHV	ORF87	UMM52765.1	39.2	74	PF00020 TNFR cysteine-rich region

[a] Sequence identity and alignment length between V511 and V516 ORFs is given
[b] Based on the ORFs of V511 the closest ORF from references genomes is given including the best matching organism, sequence identity, and alignment length. All output has been generated using eggnogmapper as described in the method section. Only results with a minimal sequence identity of 25% on amino acid level are included in the table.

Supplementary Figure S1.

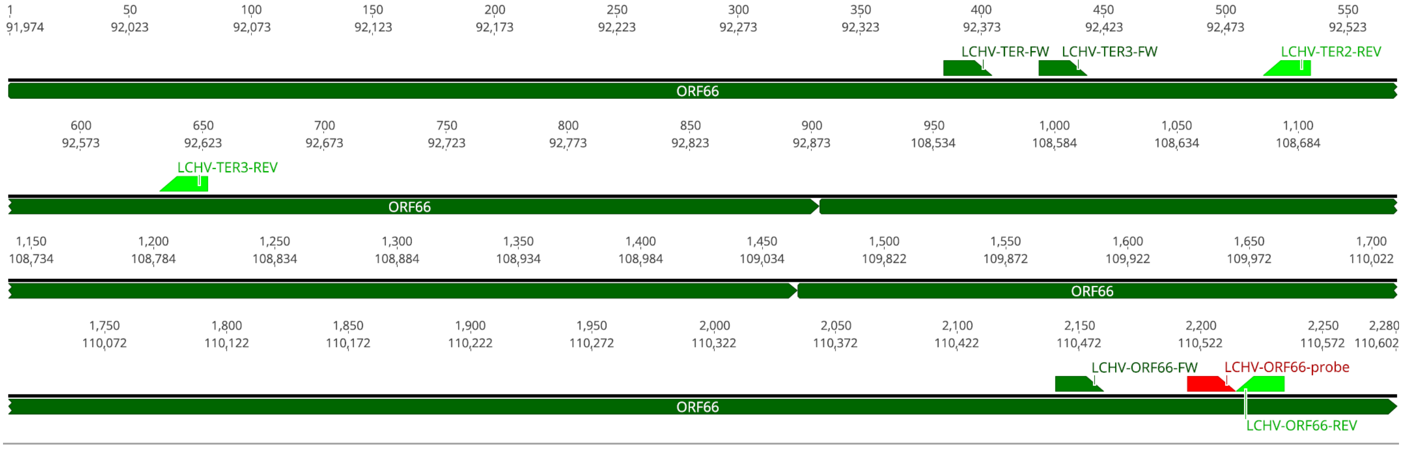

Supplementary Figure S1. Primer locations for the nested PCR as well as the qPCR designed on LCHV ORF66 (DNA packaging terminase subunit 1). The highest ruler shows the position of the spliced coding sequence, the lowest ruler shows the genomic positions. The amplicon lengths are as follows: LCHV-TER-FW - LCHV-TER3-REV = 268 bp ; LCHV-TER3-FW - LCHV-TER2-REV = 112 bp ; LCHV-ORF66-FW - LCHV-ORF66-REV = 94 bp).

Supplementary Figure S2.

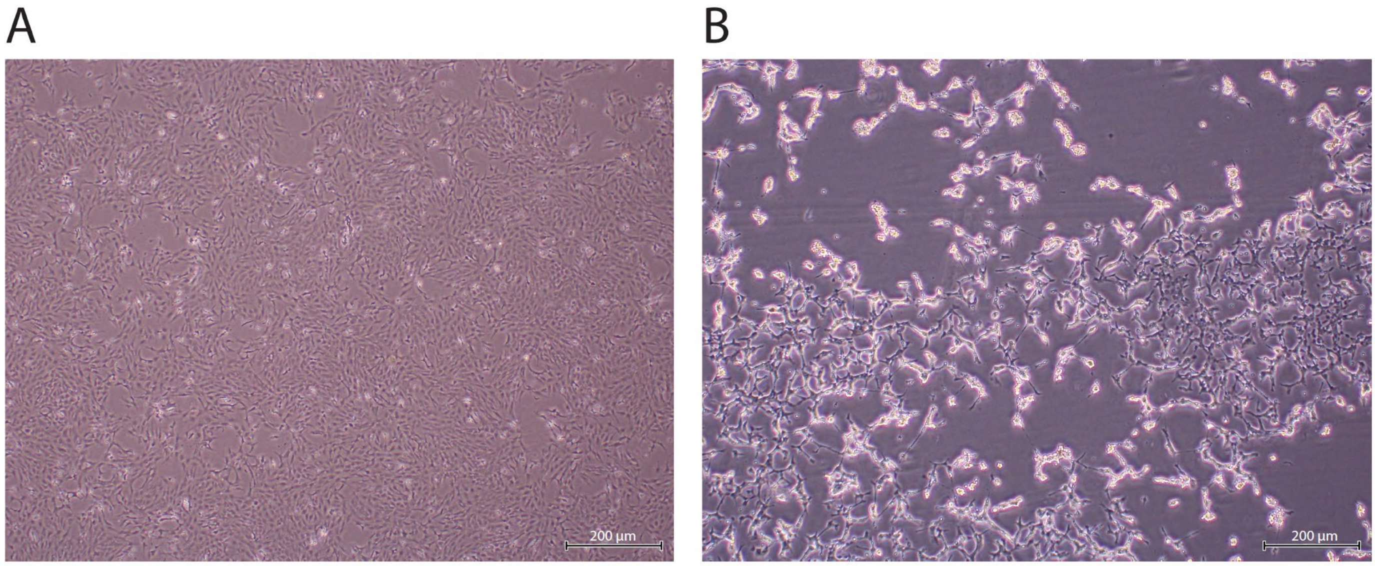

Supplementary Figure S2. (A) Morphology of SBB cells at a 90% confluent monolayer. (B) Cytopathic effect (CPE) characterized by rounding of SBB cells caused by LCHV isolate V511 72hours after infection. Pictures were captured at a 40 times magnification using an Olympus CKX41 inverted light microscope connected to a DP21 microscope camera.

Supplementary Figure S3.

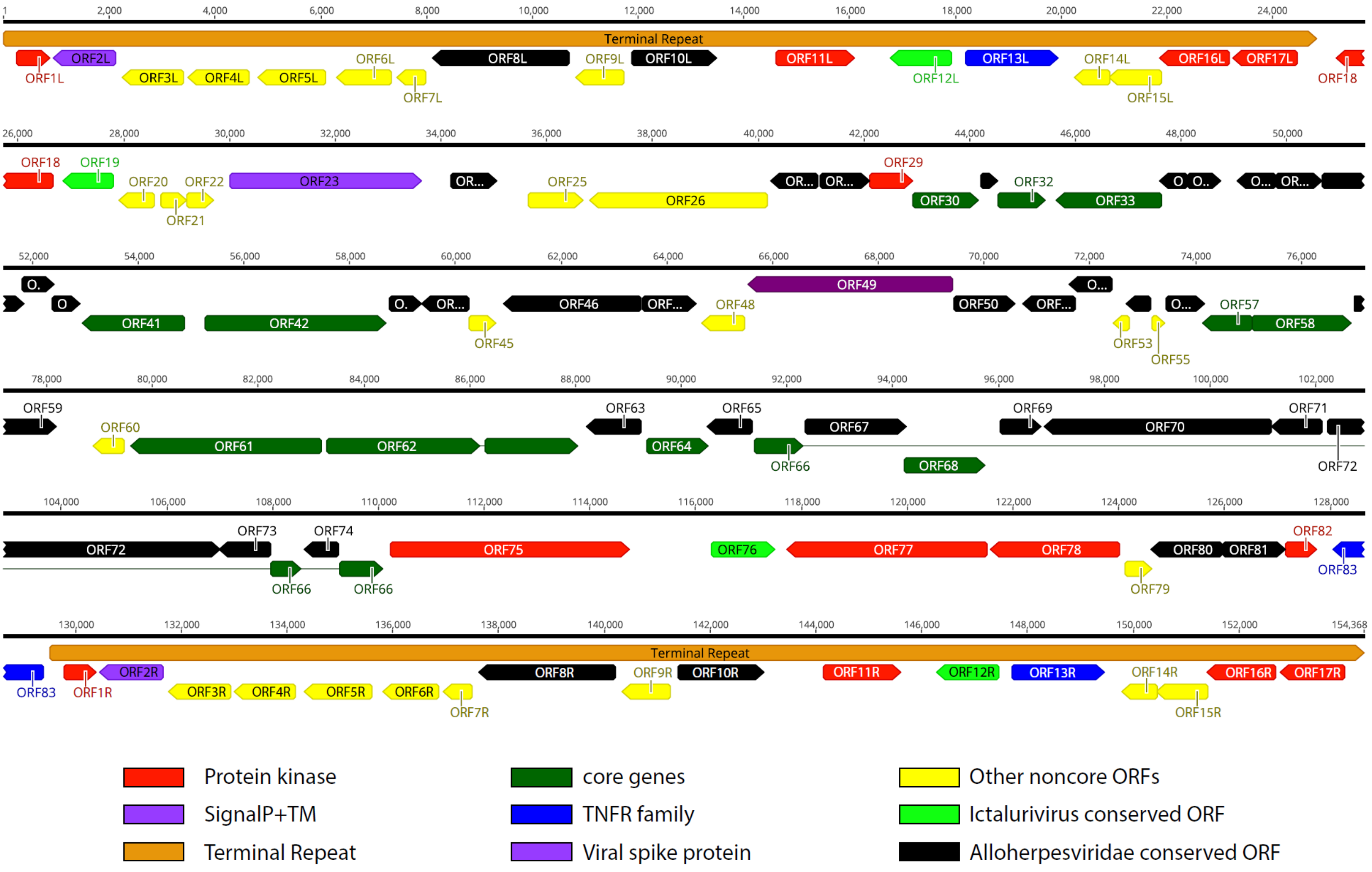

Supplementary Figure S3. Predicted functional open reading frames (ORFs) are indicated. The terminal repeats are annotated as an orange arrow box. Conservation degree and gene families are defined in the key at the bottom. Introns are depicted as thin lines connecting the exons (only (ORF62 and ORF66).

Supplementary Figure S4.

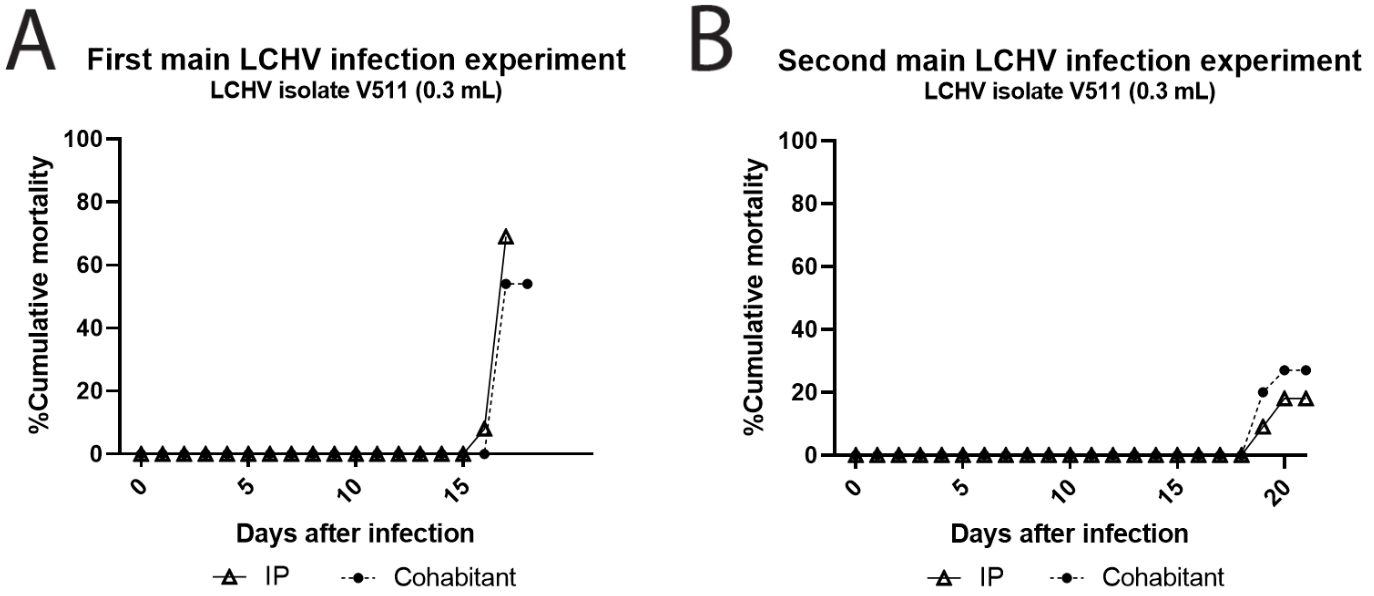

Supplementary Figure S4. These graphs show the cumulative mortality in the first (A) and second (B) main LCHV infection experiments.

Supplementary Figure S5.

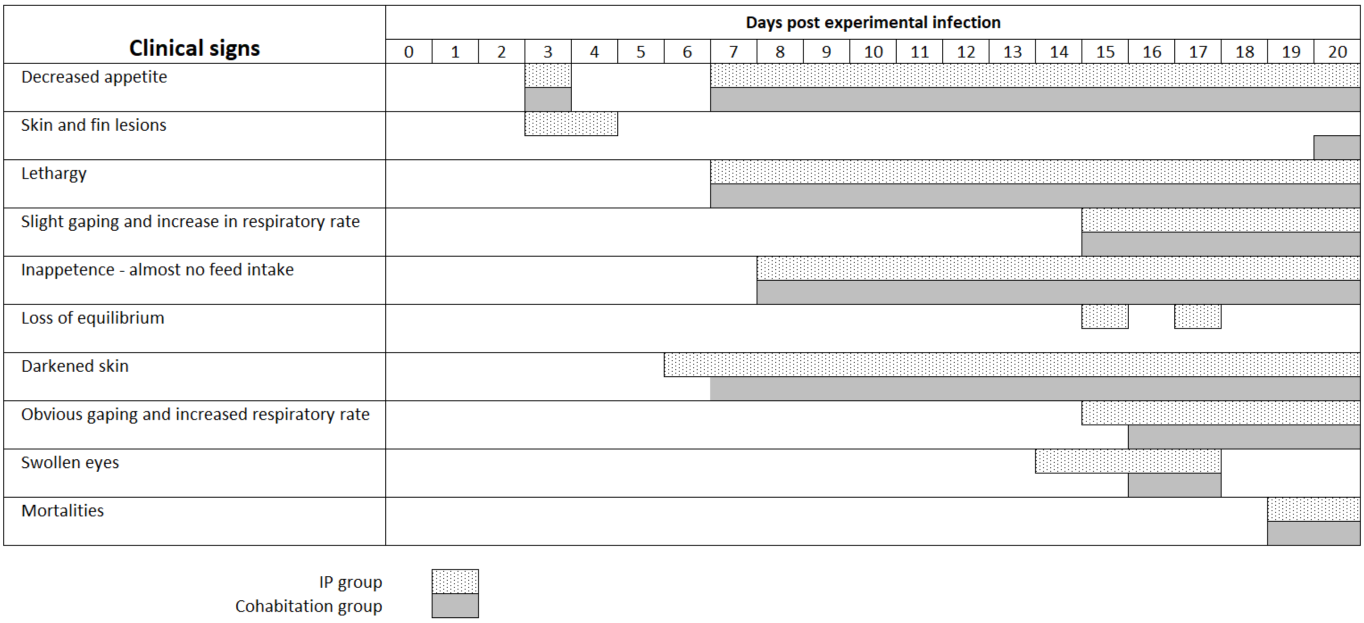

Supplementary Figure S5. Clinical signs after second main infection experiment, and natural infection in the cohabitant group.

Supplementary Figure S6.

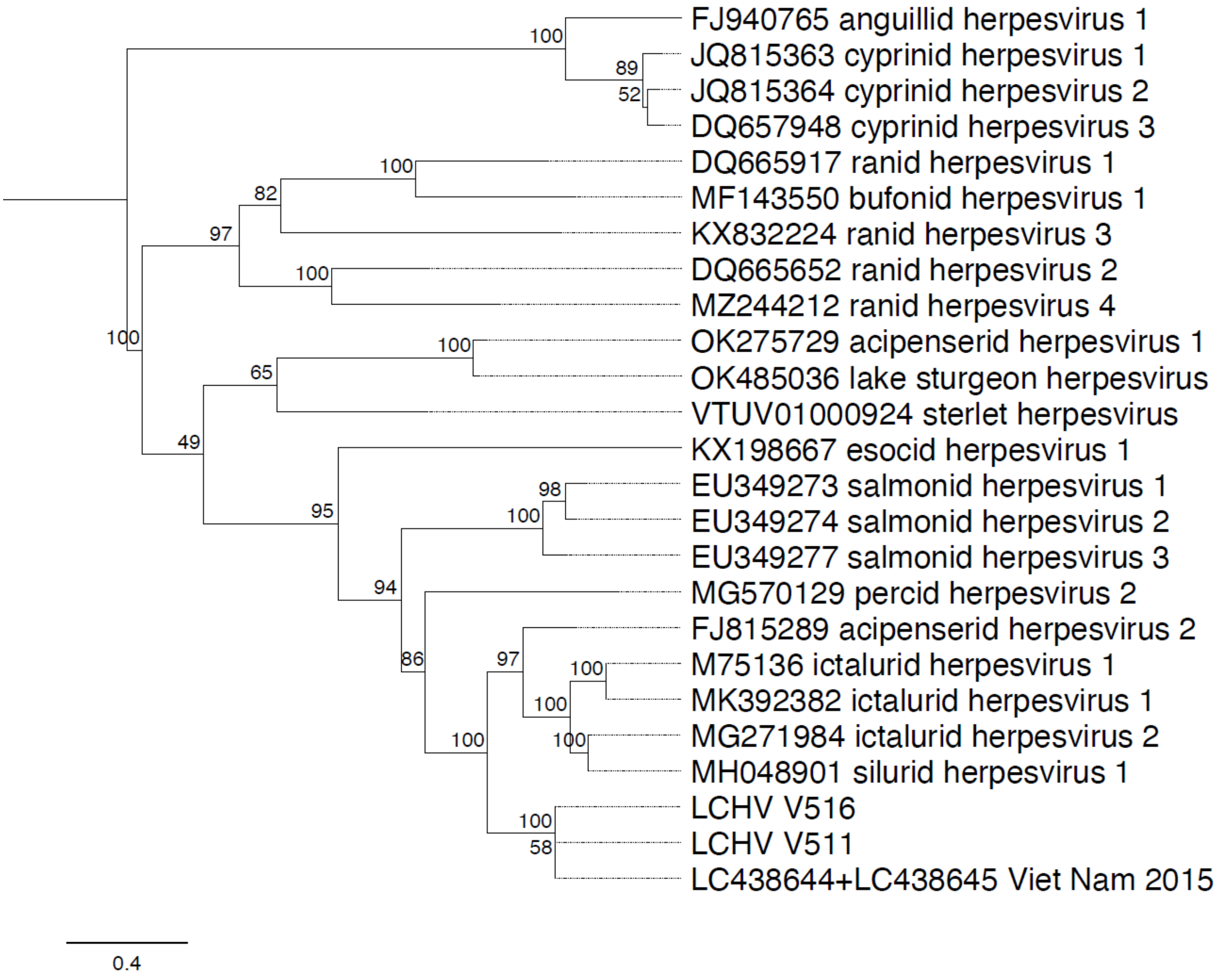

Supplementary Figure S6. Phylogenetic analysis of 22 alloherpesviruses and three sequences of LCHV isolates (V511, V516 and 'LC438644'). The partial sequences of the DNA polymerase catalytic subunit (pol) and DNA packaging terminase subunit 1 (ter1) genes were used for this analysis. The LC438644 and LC438645 sequences were obtained from Lates calcarifer infected with LCHV in Viet Nam in the year 2015: Thanasaksiri,K., Takano,R., Fukuda,K., Nguyen,H.D., Hich,T. and Sano,M.: Lates calcarifer herpesvirus gene for DNA polymerase, partial cds - Nucleotide - NCBI (nih.gov).
